# Supplementary material for: The Effects of Titanium Dioxide Nanoparticles on Osteoblasts Mineralization: A Comparison between 2D and 3D Cell Culture Models
Source: Nanomaterials (Basel). 2023 Jan 20;13(3):425. doi: 10.3390/nano13030425 (PMC9921996; doi:10.3390/nano13030425)
Supplement: Supplementary file 1 [file nanomaterials-13-00425-s001.zip › nanomaterials-2165084-supplementary.pdf]

Supplementary material

The Effects of Titanium Dioxide Nanoparticles on Osteoblasts Mineralization: A Comparison between 2D and 3D Cell Culture Models

Gabriela de Souza Castro [1,†], Wanderson de Souza [2,†], Thais Suelen Mello Lima [2], Danielle Cabral Bonfim [3], Jacques Werckmann [4], Braulio Soares Archanjo [5], José Mauro Granjeiro [2], Ana Rosa Ribeiro [6] and Sara Gemini-Piperni [1,7,*]

[1] Postgraduate Program in Odontology, Unigranrio, Duque de Caxias 25071-202, Brazil
[2] Directory of Life Sciences Applied Metrology, National Institute of Metrology Quality and Technology, Rio de Janeiro 25250-020, Brazil
[3] LabCeR Group, Federal University of Rio de Janeiro (UFRJ), Rio de Janeiro 21941-901, Brazil
[4] Visitant Professor at Brazilian Center for Research in Physics, Rio de Janeiro 22290-180, Brazil
[5] Materials Metrology Division, National Institute of Quality and Technology, Rio de Janeiro 25250-020, Brazil
[6] NanoSafety Group, International Iberian Nanotechnology Laboratory, 4715-330 Braga, Portugal
[7] Lab$_{\varepsilon}$n Group, Federal University of Rio de Janeiro (UFRJ), Rio de Janeiro 21941-901, Brazil
* Correspondence: sara.gemini@hotmail.com
† Both authors contributed equally to this investigation.

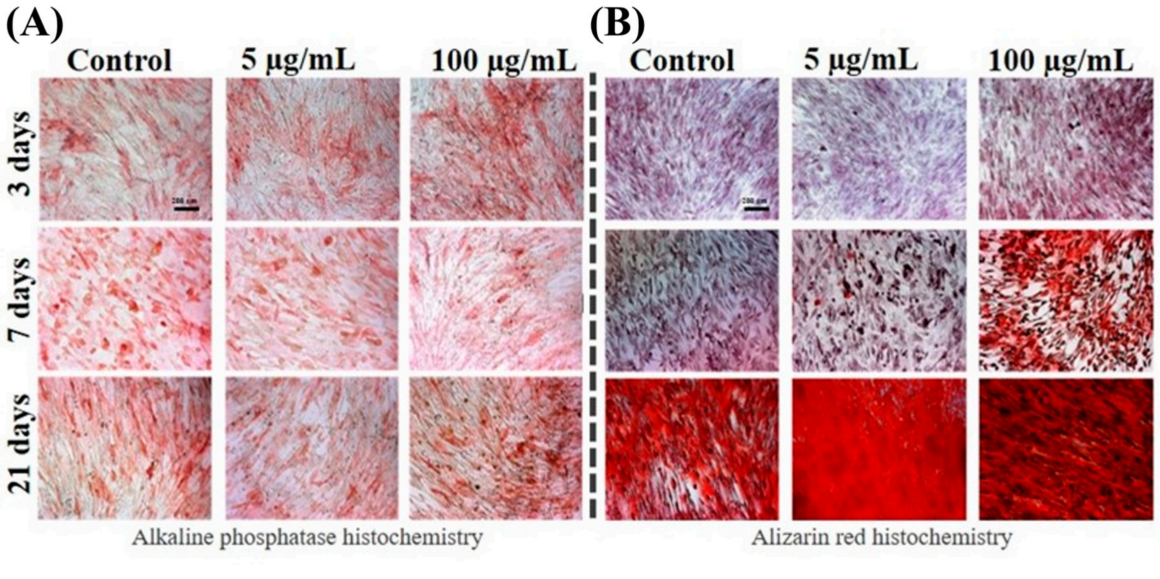

Figure S1. Primary osteoblast differentiation and mineralization: alkaline phosphatase (A) and alizarin red (B) staining in primary human osteoblasts exposed during 3, 7, and 14 days after exposure to 5 µg/mL or 100 µg/mL TiO_2 NPs. The results are representative images of three independent experiments. (scale bar: 200 µm).
